# Using cryo-EM to understand the assembly pathway of respiratory complex I

**DOI:** 10.1107/S205979832400086X

**Published:** 2024-02-19

**Authors:** Eike Laube, Jonathan Schiller, Volker Zickermann, Janet Vonck

**Affiliations:** a Department of Structural Biology, Max Planck Institute of Biophysics, 60438 Frankfurt am Main, Germany; b Institute of Biochemistry II, University Hospital, Goethe University, 60590 Frankfurt am Main, Germany; c Centre for Biomolecular Magnetic Resonance, Institute for Biophysical Chemistry, Goethe University, 60438 Frankfurt am Main, Germany; University of Leeds, United Kingdom

**Keywords:** single-particle cryo-EM, respiratory complex I, proton-pumping NADH ubiquinone oxidoreductase, complex I assembly, assembly factors

## Abstract

Complex I (proton-pumping NADH:ubiquinone oxidoreductase) is the first component of the mitochondrial respiratory chain. In recent years, high-resolution cryo-EM studies of complex I from various species have greatly enhanced the understanding of the structure and function of this important membrane-protein complex. Less well studied is the structural basis of complex I biogenesis. The assembly of this complex of more than 40 subunits, encoded by nuclear or mitochondrial DNA, is an intricate process that requires at least 20 different assembly factors in humans. These are proteins that are transiently associated with building blocks of the complex and are involved in the assembly process, but are not part of mature complex I. Although the assembly pathways have been studied extensively, there is limited information on the structure and molecular function of the assembly factors. Here, the insights that have been gained into the assembly process using cryo-EM are reviewed.

## Abstract 2

Single-particle cryo-EM is a powerful technique to study the assembly process of the largest mitochondrial respiratory chain complex, NADH:ubiquinone oxidoreductase or complex I. Here, the new insights that have been gained into the molecular functions of assembly factors are reviewed.

## 1. Introduction

Proton-pumping NADH:ubiquinone oxidoreductase is a large membrane-protein complex with a central role in aerobic energy metabolism (Galemou Yoga *et al.*, 2020 ▸; Parey *et al.*, 2020 ▸; Chung, Grba *et al.*, 2022 ▸; Sazanov, 2023 ▸). The enzyme is also called complex I because it is the first complex in the respiratory chain. With a pump stoichiometry of four protons per two electrons, complex I contributes ∼40% of the proton motive force that drives ATP synthase. Complex I dysfunction is associated with a broad spectrum of human diseases (Rodenburg, 2016 ▸; Fiedorczuk & Sazanov, 2018 ▸). The generation of reactive oxygen species by complex I is an important factor in reperfusion injury (Chouchani *et al.*, 2013 ▸).

**Figure 1:**
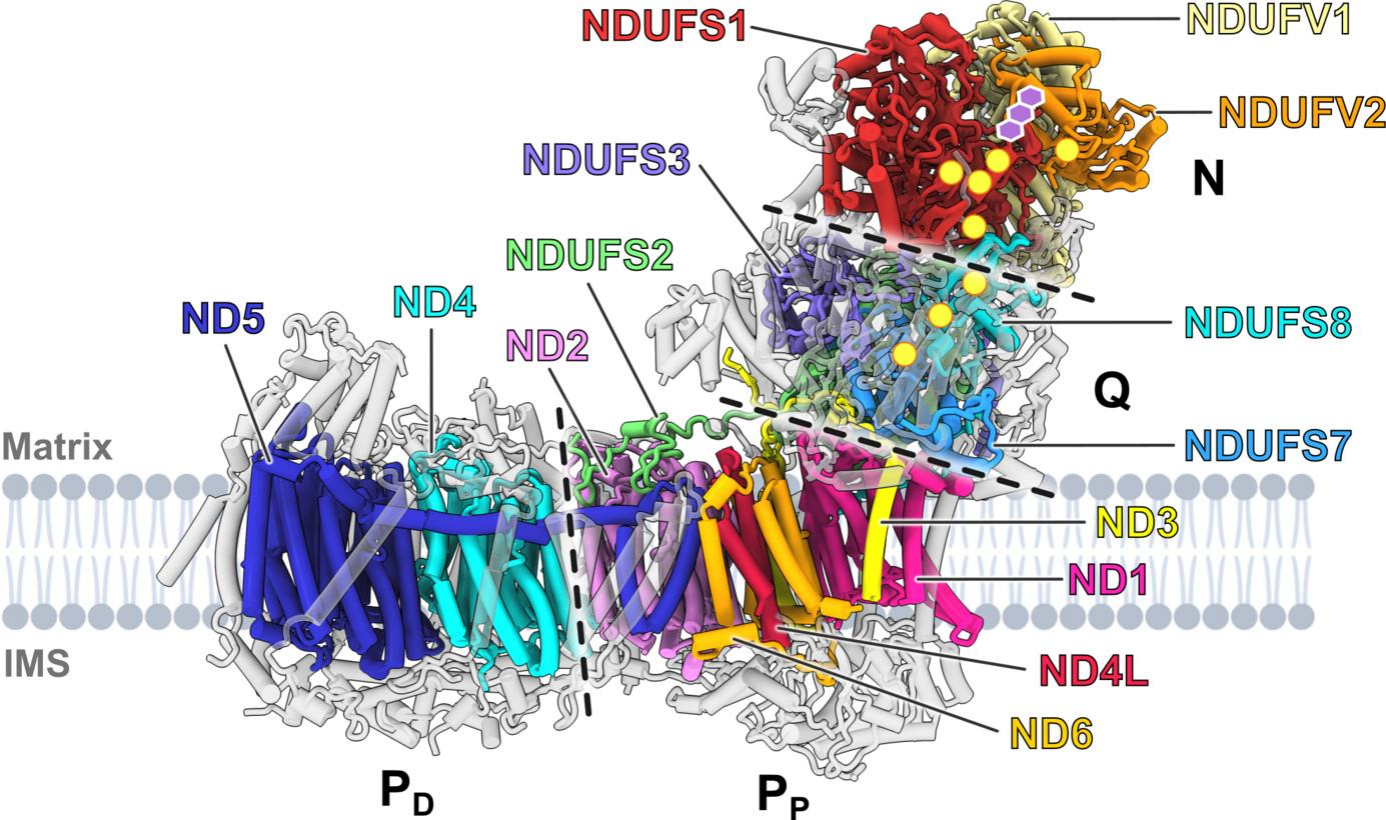
Structural overview of complex I from *Y. lipolytica*. Complex I consists of 14 conserved central subunits (shown in colour and labelled) and a varying number of accessory subunits (shown in grey) depending on the organism. The peripheral arm and the membrane arm are each formed by two modules (indicated by dashed lines). The former is composed of the N and Q modules and the latter by the proximal and distal proton-pumping modules (P_P_ and P_D_, respectively). Yellow circles indicate the positions of the iron–sulfur clusters; FMN is indicated in purple. The lipid bilayer was created with *BioRender* (https://www.biorender.com/).

Canonical complex I consists of 14 so-called central sub­units that are conserved from bacteria to humans. In some species, this number may deviate due to the fission or fusion of central subunit genes. These subunits are arranged in an L-shaped structure with a membrane arm and a peripheral arm (Fig. 1 ▸). In mitochondrial complex I, the central subunits are surrounded by accessory subunits (Stroud *et al.*, 2016 ▸; Padavannil *et al.*, 2022 ▸), the number of which is species-specific. For example, human complex I has 31 accessory subunits. When possible, we use the human complex I nomenclature (prefix NDU for NADH dehydrogenase ubiquinone) throughout this review.

Complex I can be divided into functional modules (Hunte *et al.*, 2010 ▸). The peripheral arm comprises the N module for NADH oxidation and the Q module for quinone reduction, and the membrane arm comprises the distal and proximal proton-pumping modules: P_D_ and P_P_, respectively (Fig. 1 ▸).

The first structures of complex I were determined by X-ray crystallography, initially from bacteria (Sazanov & Hinchliffe, 2006 ▸; Efremov *et al.*, 2010 ▸; Efremov & Sazanov, 2011 ▸; Baradaran *et al.*, 2013 ▸) and subsequently from mitochondria of the yeast *Yarrowia lipolytica* (Hunte *et al.*, 2010 ▸; Zickermann *et al.*, 2015 ▸). In the last decade, the cryo-EM revolution has had a huge impact on our knowledge of complex I structure, as discussed in recent reviews (Parey *et al.*, 2020 ▸; Chung, Grba *et al.*, 2022 ▸; Kampjut & Sazanov, 2022 ▸; Padavannil *et al.*, 2022 ▸). Initial cryo-EM work was focused on *Y. lipolytica* (Parey *et al.*, 2018 ▸, 2019 ▸) and mammalian species including *Bos taurus* (Zhu *et al.*, 2016 ▸; Blaza *et al.*, 2018 ▸), *Ovis aries* (Fiedorczuk *et al.*, 2016 ▸; Letts *et al.*, 2019 ▸), *Sus scrofa* (Wu *et al.*, 2016 ▸), *Mus musculus* (Agip *et al.*, 2018 ▸; Bridges *et al.*, 2020 ▸) and *Homo sapiens* (Guo *et al.*, 2017 ▸). More recently, structures have been determined from several other species, including *Escherichia coli* (Kolata & Efremov, 2021 ▸; Kravchuk *et al.*, 2022 ▸), plants (Maldonado *et al.*, 2020 ▸, 2023 ▸; Soufari *et al.*, 2020 ▸; Klusch *et al.*, 2021 ▸, 2023 ▸), thermophilic fungi (Laube *et al.*, 2022 ▸), insects (Agip *et al.*, 2023 ▸; Padavannil *et al.*, 2023 ▸) and ciliates (Zhou *et al.*, 2022 ▸; Mühleip *et al.*, 2023 ▸).

In many of these structures previously unknown components were found, showing how new regulatory or stabilizing features have been added in different species (Padavannil *et al.*, 2022 ▸; Zhou *et al.*, 2022 ▸). Many recent complex I structures are at a sufficiently high resolution (2–2.5 Å) to show the positions of functionally important water molecules, including complex I from bacteria (Kolata & Efremov, 2021 ▸; Kravchuk *et al.*, 2022 ▸), plants (Klusch *et al.*, 2023 ▸), fungi (Grba & Hirst, 2020 ▸; Parey *et al.*, 2021 ▸; Laube *et al.*, 2022 ▸) and mammals (Kampjut & Sazanov, 2020 ▸; Chung, Wright *et al.*, 2022 ▸; Gu *et al.*, 2022 ▸; Bridges *et al.*, 2023 ▸; Grba *et al.*, 2023 ▸). Another advantage of cryo-EM is that complexes with different conformations can be separated. The most conspicuous variability in complex I structures concerns the angle between the two arms, which has variously been interpreted as different steps in the catalytic cycle (Kampjut & Sazanov, 2022 ▸) or as active and deactive states (Chung, Grba *et al.*, 2022 ▸). High-resolution structures of these two states revealed more subtle differences, including the conformation of catalytically important loops in the Q-binding cavity, different positions of quinone in this cavity and a switch between an α- and π-helical conformation of TMH3^ND6^ that appears to control the continuity of a water chain in the membrane arm. Clues to the roles of specific residues or subunits can be gained by structural analysis of disease mutants in model animals (Yin *et al.*, 2021 ▸, 2024 ▸) or genetically modified complex I (Cabrera-Orefice *et al.*, 2018 ▸; Parey *et al.*, 2019 ▸; Galemou Yoga *et al.*, 2020 ▸). The latter requires a genetically accessible species. The yeast *Y. lipolytica* has long been established as a model organism for complex I research (Ahlers *et al.*, 2000 ▸; Kerscher *et al.*, 2001 ▸; Abdrakhmanova *et al.*, 2004 ▸; Morgner *et al.*, 2008 ▸), while for metazoan complex I, *Drosophila melanogaster* has recently been introduced (Agip *et al.*, 2023 ▸; Padavannil *et al.*, 2023 ▸).

While the structure of complex I is now known in great detail, the structural basis of complex I biogenesis has been much less explored. Early work on complex I assembly was based on gene disruption in the model organism *Neurospora crassa* (Tuschen *et al.*, 1990 ▸; Nehls *et al.*, 1992 ▸; Küffner *et al.*, 1998 ▸). Later, the detection of assembly intermediates in mitochondria from patients with complex I defects provided valuable information on complex I biogenesis in humans (Antonicka *et al.*, 2003 ▸; Lazarou *et al.*, 2007 ▸; Vogel, van den Brand *et al.*, 2007 ▸). Furthermore, it became increasingly clear that pathogenic complex I defects are frequently not associated with changes in structural genes, but with mutations in genes encoding so-called assembly factors (Nouws *et al.*, 2012 ▸; Pagniez-Mammeri *et al.*, 2012 ▸). Assembly factors are proteins that are involved in the biogenesis of a complex, but are not part of the final enzyme. To date, more than 20 assembly factors have been described for human complex I (Table 1 ▸). In recent years, complexome profiling (Heide *et al.*, 2012 ▸) and proximity-labelling methods have led to a detailed description of complex I biogenesis (Guerrero-Castillo *et al.*, 2017 ▸; Formosa *et al.*, 2020 ▸). It is now clear that the assembly of mitochondrial complex I is a stepwise process that mirrors its modular architecture, as was first suggested by Ugalde *et al.* (2004 ▸). However, despite all of these impressive advances (Garcia *et al.*, 2017 ▸; Guerrero-Castillo *et al.*, 2017 ▸; Ligas *et al.*, 2019 ▸; Formosa *et al.*, 2020 ▸), the structures of assembly factors and their molecular functions have only recently started to emerge.

The cryo-EM revolution has now opened new avenues for studying complex I assembly and assembly factors in molecular detail. In contrast to X-ray crystallography, cryo-EM requires only small amounts of material and can deal with heterogeneous samples, which are advantages for the characterization of short-lived intermediates. Recently, we and others have used cryo-EM to understand the structure of complex I assembly intermediates and infer assembly factors in *Y. lipolytica* (Parey *et al.*, 2019 ▸; Schiller *et al.*, 2022 ▸), humans (Giachin *et al.*, 2021 ▸; McGregor *et al.*, 2023 ▸), mice (Yin *et al.*, 2024 ▸) and plants (Soufari *et al.*, 2020 ▸). Taken together, these studies show that cryo-EM is a powerful tool to gain structural insights into the assembly pathways of complex I.

## 2. Assembly intermediates associated with NDUFAF1

**Figure 2:**
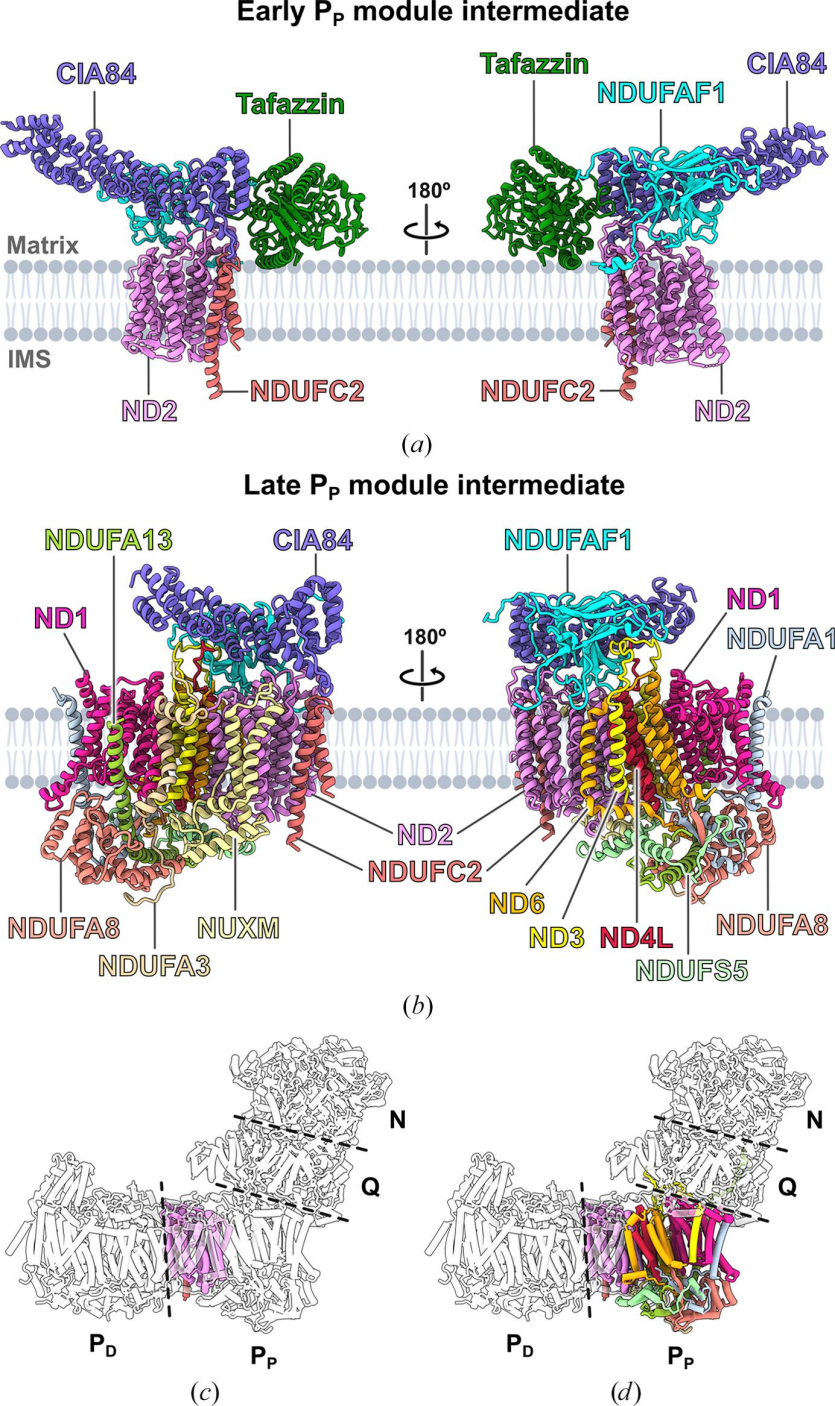
Cryo-EM structures of complex I assembly intermediates. (*a*) The smaller assembly intermediate of the P_P_ module of complex I from *Y. lipolytica*, referred to as the early P_P_ module intermediate, is composed of the subunits ND2 and NDUFC2, the assembly factors CIA84 and NDUFAF1, and the acyltransferase tafazzin. (*b*) The second intermediate (the late P_P_ module intermediate) contains all of the subunits of the P_P_ module of complex I. CIA84 and NDUFAF1 retain their structure and binding sites, but tafazzin is no longer bound. (*c*, *d*) Location of the modules in *Y. lipolytica* complex I with subunits of the early (*c*) and late (*d*) P_P_ module intermediate coloured. The lipid bilayer was created with *BioRender*.

The assembly factor NDUFAF1 is highly conserved in fungi, plants and animals, and is an essential part of the so-called mitochondrial complex I intermediate assembly (MCIA) complex in humans (Heide *et al.*, 2012 ▸; Elurbe & Huynen, 2016 ▸; Formosa *et al.*, 2018 ▸). The MCIA complex is critical for the assembly process of the P_P_ module of complex I (Formosa *et al.*, 2020 ▸). Deletion of the *ndufaf1* gene in the fungus *Y. lipolytica* strongly impairs the assembly process of mitochondrial complex I, while in the *ndufaf1Δ* strain only the P_D_ module was fully assembled (Schiller *et al.*, 2022 ▸), in agreement with the crucial role of NDUFAF1 in assembly of the P_P_ module (Fig. 2 ▸ *d*). The absence of the Q and N modules indicates that there must be a regulatory mechanism that prevents the accumulation of peripheral arm intermediates that cannot be processed further because the P_P_ module is lacking. Notably, this dependence seems to be less pronounced in other fungal species such as *N. crassa*, where *ndufaf1Δ* strains show fully assembled peripheral arms of complex I (Küffner *et al.*, 1998 ▸).

In order to gain structural insight into NDUFAF1 and its assembly intermediates, a *Y. lipolytica* mutant was generated in which an *ndufaf1* deletion was complemented with an NDUFAF1 variant carrying a C-terminal twin Strep-tag (Schiller *et al.*, 2022 ▸). After the purification of NDUFAF1 and NDUFAF1-associated assembly intermediates, samples were analysed by cryo-EM. Two distinct complexes were found with molecular weights of ∼170 and ∼280 kDa, and cryo-EM maps of both complexes were obtained at a resolution of ∼3.2 Å (Figs. 2 ▸ *a* and 2 ▸ *b*). The smaller complex represents an early-stage assembly intermediate of the P_P_ module and is formed by the P_P_ module subunits ND2 and NDUFC2, the assembly factor NDUFAF1 and the fungus-specific assembly factor CIA84 (Figs. 2 ▸ *a* and 2 ▸ *c*). Unexpectedly, the ∼43 kDa large acyltransferase tafazzin is also an integral part of the early P_P_ module intermediate in *Y. lipolytica*, which will be discussed below (Schiller *et al.*, 2022 ▸).

**Figure 3:**
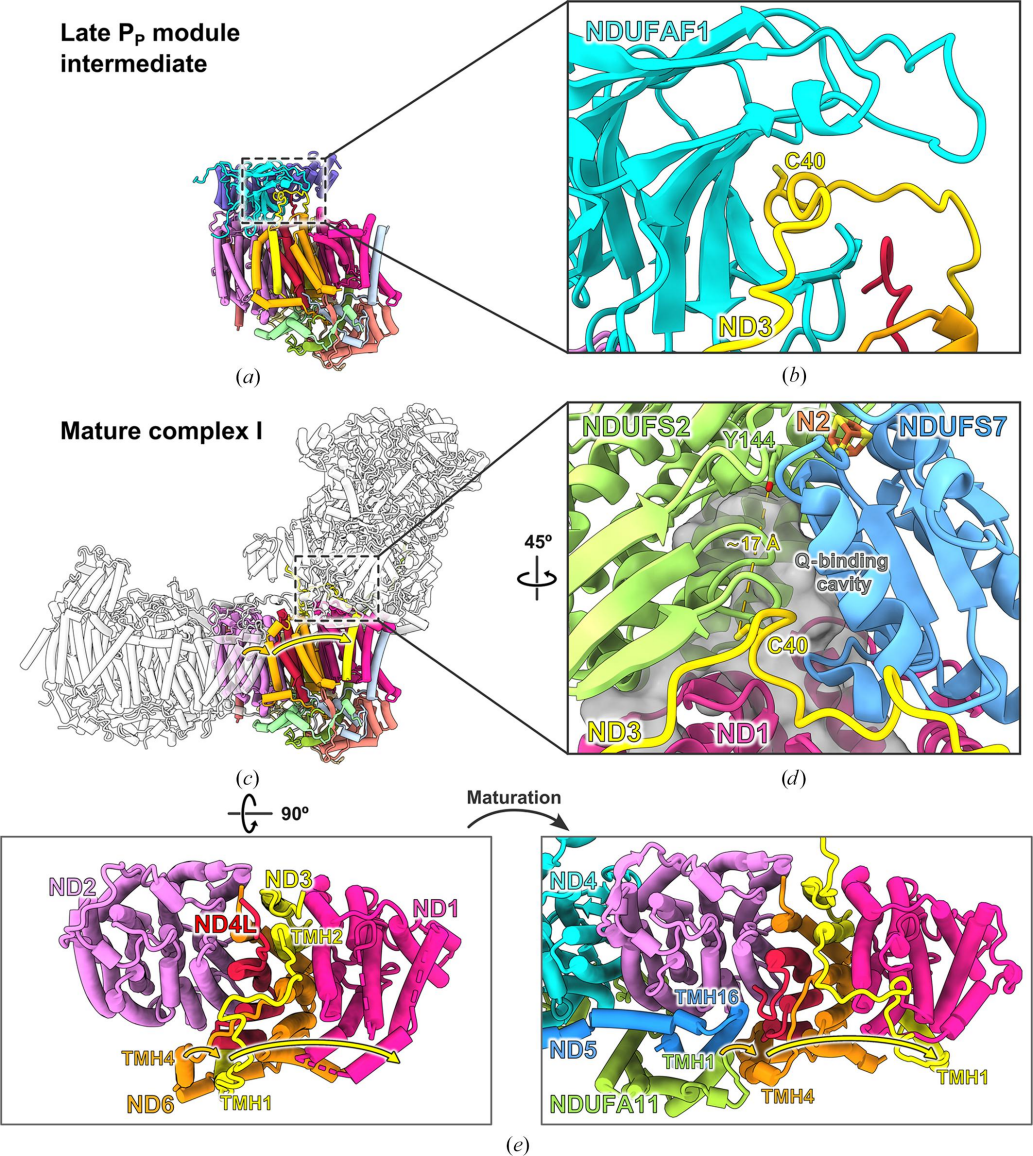
The role of NDUFAF1 in the assembly process of complex I from *Y. lipolytica*. (*a*, *b*) In the late P_P_ module intermediate, the TMH1–2 loop of the ND3 subunit with its conserved cysteine is bound in a binding cleft of NDUFAF1. (*c*, *d*) In mature complex I, the same loop is bound at the interface between the membrane and peripheral arms, mainly formed by the central subunits NDUFS2, NDUFS7 and ND1. In this conformation the cysteine is close to the Q-binding cavity (∼6 Å) and the conserved Tyr144^NDUFS2^ (∼17 Å), a residue critical for binding ubiquinone near Fe–S cluster N2 (Tocilescu *et al.*, 2010 ▸). The conformational change of the TMH1–2 loop of ND3 upon complex I maturation is accompanied by major shifts of TMH1^ND3^ and TMH4^ND6^ (yellow and orange arrows, respectively). (*e*) View from the matrix side of the P_P_ module. TMH1^ND3^ and TMH4^ND6^ change their positions upon maturation as their binding sites in the assembly intermediate (left) overlap with the binding sites of TMH16^ND5^ and TMH1^NDUFA11^ of the P_D_ module (right). Assembly factors and accessory subunits, except NDUFA11, are omitted for clarity. (*e*) is adapted from Schiller *et al.* (2022 ▸).

In the larger complex, named the late P_P_ module intermediate, the remaining four central subunits and six accessory subunits of the P_P_ module have been added (Figs. 2 ▸ *b* and 2 ▸ *d*). However, several subunits in the late P_P_ module intermediate still differ in their conformation from the mature complex. This is evident in particular in subunit ND3, the long TMH1–2 loop of which is held in a binding cleft of assembly factor NDUFAF1 (Figs. 2 ▸ *b* and 3 ▸ *a*). ND3 has an unusual topology, with TMH1 and TMH2–3 located on opposite sides of the membrane arm, ∼35 Å apart (Fig. 3 ▸ *e*). In mature complex I, TMH1^ND3^ is located near ND1, but in the assembly intermediate it is shifted by ∼40 Å to a more distal position near subunits ND4L and ND6, where it occupies the position taken by TMH4^ND6^ after the completion of assembly (Fig. 3 ▸ *e*). In mature complex I the TMH1–2^ND3^ loop interacts with the peripheral arm (Fig. 3 ▸ *d*) and is thought to play a critical role in catalysis (Cabrera-Orefice *et al.*, 2018 ▸). It contains a conserved cysteine close to the ubiquinone-binding cavity (Fig. 3 ▸ *d*) that is buried in the binding cleft of NDUFAF1 in the assembly intermediate (Fig. 3 ▸ *b*).

### 2.1. The role of the assembly factor NDUFAF1

The structure of the late P_P_ module intermediate suggests at least two functions of NDUFAF1. Firstly, it ensures an assembly-specific position of TMH1^ND3^. This is necessary because in mature complex I, TMH1 and the TMH1–2 loop of ND3 interact with ND1 and the Q module, respectively (Fig. 3 ▸), neither of which are present when the ND3 subunit attaches to the nascent P_P_ module. Thus, NDUFAF1 secures the loop and TMH1^ND3^ in an assembly-specific arrangement until all of the remaining subunits of the P_P_ module have been added. Secondly, since chemical modifications of the conserved cysteine in the TMH1–2 loop of ND3 strongly impair complex I activity (Galkin *et al.*, 2008 ▸; Blaza *et al.*, 2018 ▸; Cabrera-Orefice *et al.*, 2018 ▸), the shielding of the cysteine in the binding cleft of NDUFAF1 (Fig. 3 ▸ *b*) may have an important protective function during the assembly process.

**Figure 4:**
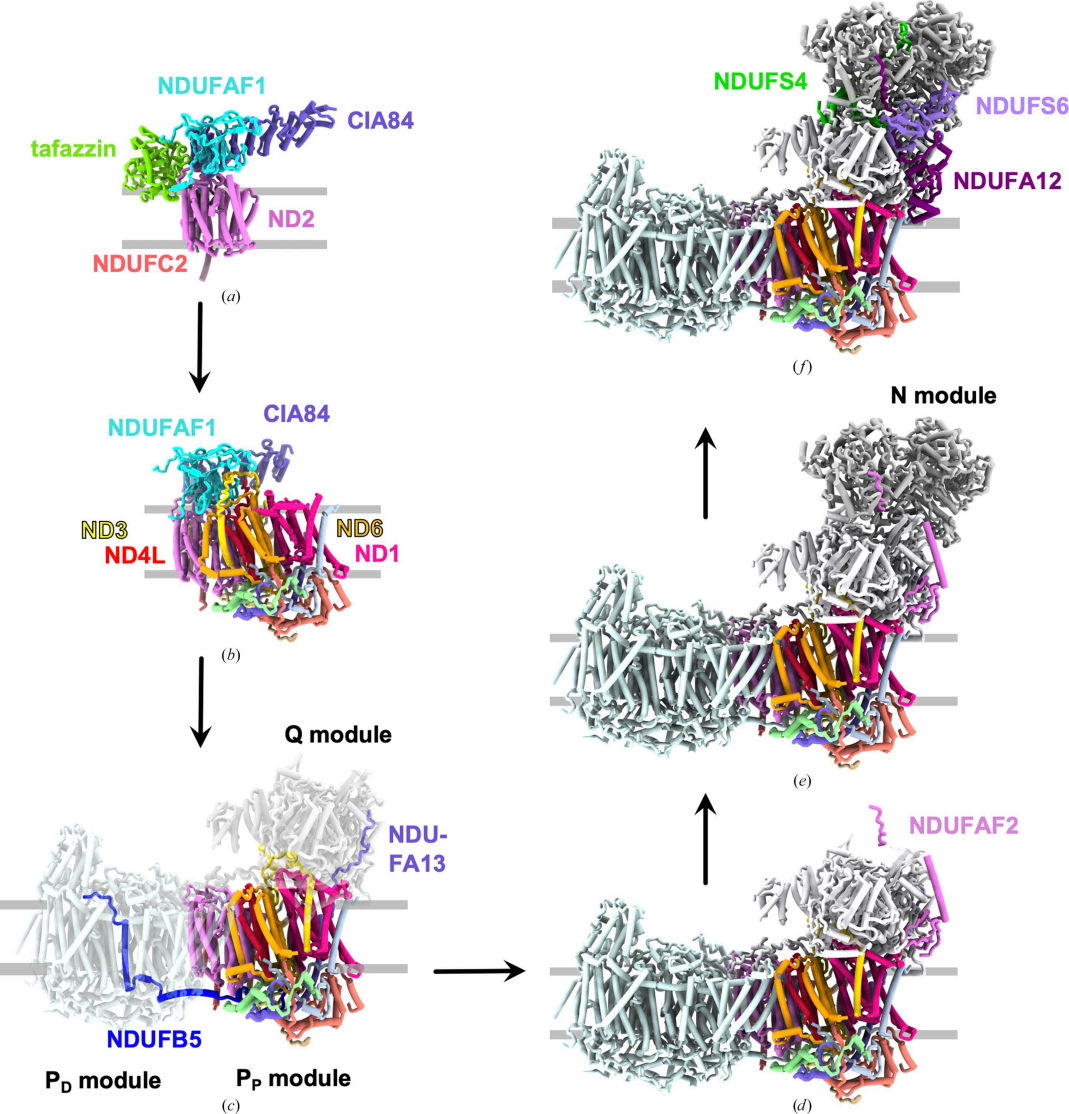
The assembly pathway of complex I in *Y. lipolytica*. (*a*) The central subunit ND2 and accessory subunit NDUFC2 associate with the assembly factors NDUFAF1 and CIA84 and with tafazzin to form the early P_P_ module intermediate. (*b*) The central membrane subunits ND4L, ND3, ND6 and ND1 are added successively, whereby the TMH1–2 loop of ND3 is secured by NDUFAF1. The addition of the accessory subunits NDUFA1, NDUFA3, NDUFA8, NDUFA13 (one TMH each), NUXM (two TMHs) and NDUFS5 (IMS side) completes the late P_P_ module intermediate. (*c*) The C-terminal domain of NDUFB5 on the IMS side makes a large contribution to joining the P_D_ and P_P_ modules. The core of the Q module is anchored by the N-terminal coil of NDUFA13. The assembly factors are released and ND3, ND6 and ND1 rearrange to accommodate the Q-module interaction partners. (*d*) The assembly factor NDUFAF2 attaches to the Q module. (*e*) The N module binds to the Q module, interacting with the C-terminal part of NDUFAF2. (*f*) The combined action of NDUFS4, NDUFS6 and NDUFA12 releases the assembly factor, leading to mature complex I.

As mentioned, in the late P_P_ module intermediate TMH1^ND3^ occupies the position of TMH4^ND6^ in mature complex I, and the latter helix is displaced towards the ND2 subunit. However, this assembly-specific position overlaps with TMH16^ND5^ and TMH1^NDUFA11^ of the P_D_ module in mature complex I. Thus, the shift of TMH1^ND3^ and TMH4^ND6^ to their mature positions is either associated with addition of the P_D_ module to the P_P_ module or must occur before this assembly event (Fig. 3 ▸ *e*). Furthermore, the structure of the late P_P_ module intermediate provides clues about later steps in complex I assembly. The conformation of the accessory subunits on the intermembrane space (IMS) side of the P_P_ module is identical to that in mature complex I, and they form a cleft into which the C-terminus of the NDUFB5 accessory subunit of the P_D_ module binds in mature complex I (see Fig. 4 ▸ *c*). NDUFB5 is firmly anchored in the P_D_ module and is suggested to play a role in joining the two subdomains (P_P_ and P_D_) of the P module (Schiller *et al.*, 2022 ▸). This anchoring role of NDUFB5 has also been suggested in mammalian complex I (Soufari *et al.*, 2020 ▸; Padavannil *et al.*, 2022 ▸). In plants, which lack NDUFB5, a similar role has been proposed for the plant-specific subunit P1 (Soufari *et al.*, 2020 ▸; Padavannil *et al.*, 2022 ▸; Meyer *et al.*, 2022 ▸). Furthermore, the NDUFA13 subunit consists of a long, kinked helix, half of which lies on the IMS side, while the other half spans the membrane. Its N-terminus forms a coil that is disordered in the assembly intermediate, but runs along the side of NDUFS2 of the Q module in mature complex I (Fig. 4 ▸ *c*). NDUFA13 is thus suggested to play a role in anchoring the Q and P_P_ modules (Schiller *et al.*, 2022 ▸).

### 2.2. The fungus-specific assembly factor CIA84

**Figure 5:**
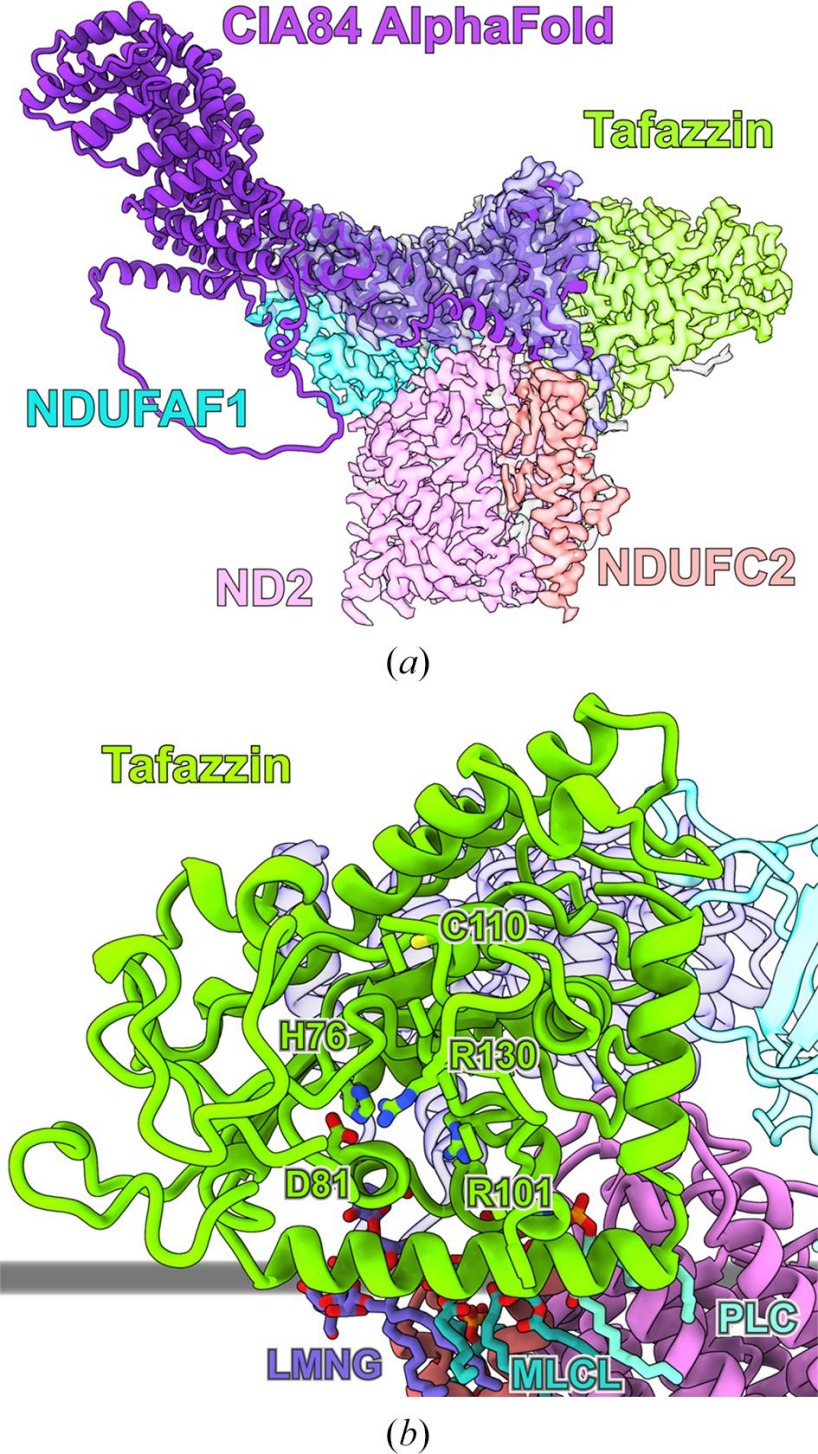
The role of CIA84 and tafazzin in the early P_P_ module assembly intermediate. (*a*) Cryo-EM density of the early P_P_ module assembly intermediate of *Y. lipolytica* (coloured by subunit as in Fig. 2 ▸ *a*) with a fitted *AlphaFold* prediction of CIA84 (purple ribbon). (*b*) The structure of tafazzin. Conserved active-site residues and lipids found in the cryo-EM structure are indicated. The membrane surface is indicated in grey.

CIA84 is a 97 kDa protein with structural similarities to the pentatricopeptide-repeat protein (PPR) family. PPR proteins consist of serial repeats of 35-residue helix–turn–helix motifs that together typically form a superhelical structure. PPR proteins are widespread in plants, where they are typically involved in RNA binding (Manna, 2015 ▸). However, despite the structural similarities to PPRs, CIA84 does not contain the sequence motifs that are associated with RNA binding (Schiller *et al.*, 2022 ▸). In the P_P_ module assembly intermediates only the C-terminal half of CIA84 is resolved. This region of CIA84 makes connections to all other proteins of the early P_P_ module intermediate and appears to function as a core that ensures the binding of NDUFAF1 and tafazzin to ND2 and NDUFC2. In the late P_P_ module intermediate, CIA84 also binds the C-terminus of ND4L. This interaction keeps the C-terminus in an elevated position, making room for the matrix loop of ND6 and the aforementioned TMH1–2 loop of ND3, which can thus assume their assembly-specific positions (Fig. 3 ▸ *e*). The function of the C-terminal 422 amino acids of CIA84 can be summarized as the securing of assembly factors of the early intermediate and the facilitation of the assembly-specific conformation of ND4L (Schiller *et al.*, 2022 ▸). The first 430 residues are unresolved in the cryo-EM structures, probably due to high flexibility. *AlphaFold* predictions also indicate PPR-like structures in this region (Fig. 5 ▸ *a*). The role of this part of CIA84 is presently unknown.

### 2.3. Tafazzin is associated with a complex I assembly intermediate in *Y. lipolytica*

The 3.2 Å resolution cryo-EM map of the early P_P_ module intermediate of *Y. lipolytica* contained extra density on the matrix side that could not be assigned to known complex I subunits or assembly factors. The high quality of the map made it possible to recognize short sequence fragments, and the protein was identified as the cardiolipin-remodelling enzyme tafazzin (Schiller *et al.*, 2022 ▸). The association of tafazzin with a complex I assembly intermediate is in agreement with complexome profiling data of *Y. lipolytica* mitochondria (Alfredo Cabrera-Orefice, personal communication). Cardiolipin is the key lipid of the inner mitochondrial membrane; its remodelling by tafazzin results in a species- or organ-specific acyl-chain composition with a high proportion of unsaturated lipids that is essential for the proper activity of OXPHOS complexes and for cristae structure (Schlame, 2013 ▸; Wang, Li *et al.*, 2020 ▸). Tafazzin is highly conserved throughout eukaryotes and in humans; mutations in its gene are the cause of Barth syndrome, a severe disease that typically presents as cardiomyopathy, muscle weakness and neutropenia (Bione *et al.*, 1996 ▸; Schlame, 2013 ▸).

Tafazzin transfers acyl chains from phospholipids to lysophospholipids and belongs to the acyltransferase family (Xu *et al.*, 2006 ▸); however, its structure had not previously been determined. The active site contains the conserved residues His76 and Asp81, and several conserved arginine residues are involved in binding the phospholipid substrates (Fig. 5 ▸ *b*). Tafazzin interacts with the membrane via an amphipathic helix and the active site is oriented towards the membrane, with access to its substrates (Figs. 2 ▸ *a* and 5 ▸ *b*; Schiller *et al.*, 2022 ▸). For this orientation, the association with the assembly intermediate appears to be critical. Tafazzin makes tight contacts with ND2 and NDUFAF1 as well as CIA84. However, all interactions are mediated by a 30-residue loop that is not conserved, even in other fungal species such as *N. crassa* and *Pichia pastoris*. Thus, the observed interaction of tafazzin with a complex I assembly intermediate (Schiller *et al.*, 2022 ▸) does not appear to be a general phenomenon. Interestingly, in insect or mammalian cells tafazzin has been shown to be associated with multiple protein complexes, but their identity is largely unclear (Claypool *et al.*, 2008 ▸; Xu *et al.*, 2015 ▸). The recruitment of tafazzin near the biogenesis site of OXPHOS complexes may be beneficial to ensure a sufficient supply of cardiolipin when and where it is needed.

## 3. The human MCIA complex

**Figure 6:**
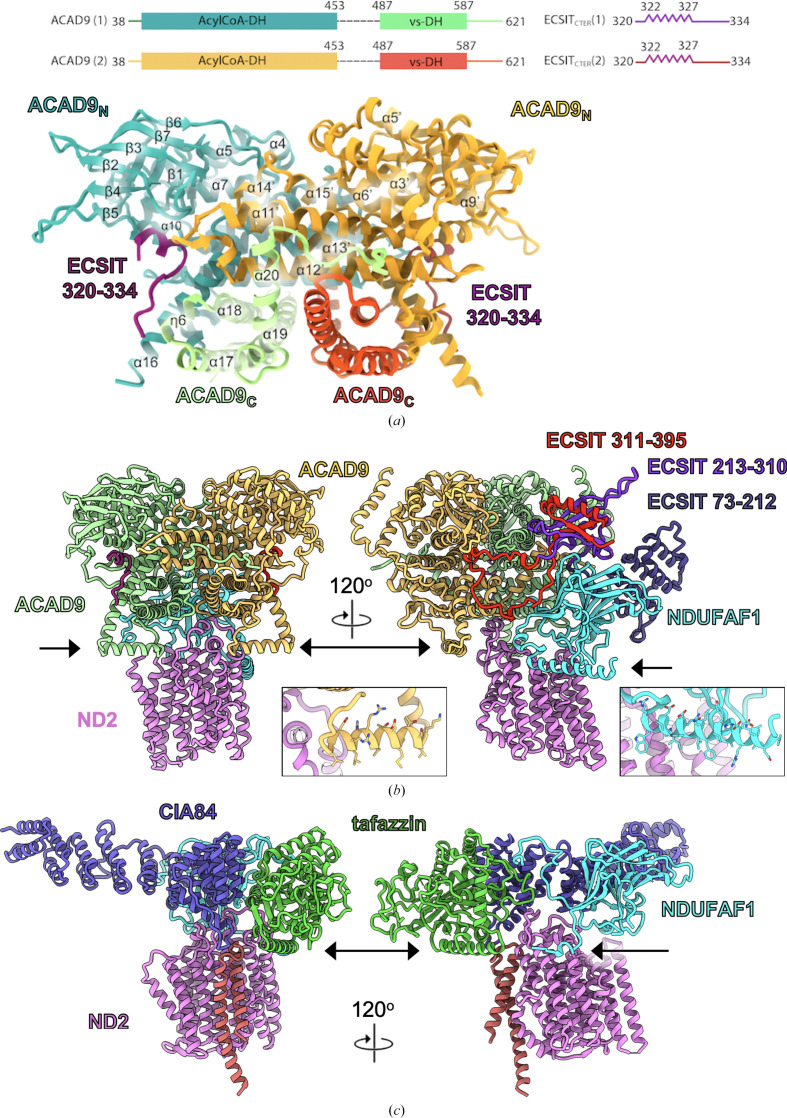
The human MCIA complex. (*a*) Cryo-EM structure of the human ACAD9–ECSIT_CTER_ complex, formed *in vitro* from subunits expressed in *E. coli*, with a schematic of the sequences of ACAD9 and ECSIT that were modelled in the EM map. Reprinted from McGregor *et al.* (2023 ▸). (*b*) *AlphaFold-Multimer* prediction of human ACAD9 (two copies), ECSIT (two copies), NDUFAF1 and ND2 in two different orientations. The left panel shows only ECSIT residues 320–334 that were modelled in the EM structure; the right panel shows the high-confidence prediction of one copy. Black arrows indicate amphipathic membrane anchors. The insets show the amphipathic helices of ACAD9 (460–475, yellow) and NDUFAF1 (81–106, cyan). (*c*) The *Y. lipolytica* early P_P_ module intermediate in the same orientations aligned on ND2.

With the exception of NDUFAF1, a different set of assembly factors are involved in formation of the P_P_ module in mammals than in yeast (Table 1 ▸). The MCIA complex consists of the matrix-assembly factors NDUFAF1, ACAD9 and ECSIT, which are all essential for forming the first assembly intermediate (Guerrero-Castillo *et al.*, 2017 ▸; Formosa *et al.*, 2020 ▸), and the transmembrane subunits COA1, TMEM126B and TMEM186, which are added later (Guerrero-Castillo *et al.*, 2017 ▸; Formosa *et al.*, 2020 ▸). The MCIA complex is responsible for the formation of the mammalian P_P_-b module, which includes ND2, ND4L, ND3 and ND6. The recently identified assembly factor sideroflexin 4 (SFXN4), a transmembrane protein related to serine transporters, appears to be involved in the insertion of ND6 (Jackson *et al.*, 2022 ▸). Although there is as yet no structural information about the MCIA complex in a membrane context, recent structural studies have focused on the ACAD9–ECSIT subcomplex (Giachin *et al.*, 2021 ▸; Xia *et al.*, 2021 ▸; McGregor *et al.*, 2023 ▸). ACAD9 is an acyl-CoA dehydrogenase (ACAD) enzyme and is closely related to the very long chain acyl-CoA dehydrogenase (VLCAD) involved in the fatty-acid β-oxidation (FAO) pathway (Nouws *et al.*, 2010 ▸). FAO activity in ACAD enzymes relies on a flavin adenine dinucleotide (FAD) cofactor. ECSIT binding to ACAD9 was found to trigger loss of FAD, switching it from an FAO enzyme to a complex I assembly factor (Giachin *et al.*, 2021 ▸; Xia *et al.*, 2021 ▸). Cryo-EM structures have been described of *in vitro*-produced human complexes of ACAD9 with the C-terminal domain of ECSIT (ECSIT_CTER_), first at low resolution (Giachin *et al.*, 2021 ▸) and recently at 3 Å resolution (McGregor *et al.*, 2023 ▸). The structures show ACAD9 as a dimer with an ∼20-residue loop of ECSIT bound to each monomer (Fig. 6 ▸ *a*). Comparison with a cryo-EM map of an ACAD9 dimer alone shows that the binding of ECSIT to ACAD9 induces conformational changes that cause the release of FAD (McGregor *et al.*, 2023 ▸). However, most of the structure of ECSIT remains unresolved and the ACAD9–ECSIT_CTER_ structure does not provide hints about the function of ACAD9 and ECSIT in complex I assembly. A recent complex I cryo-EM structure of a mouse disease mutant showed a bound ACAD dimer (Yin *et al.*, 2024 ▸). This was, however, identified as the FAO enzyme ACADVL instead of ACAD9, and moreover the authors concluded that the association is nonphysiological and is a cross-linking artefact (Yin *et al.*, 2024 ▸).

A minimal first assembly intermediate of the human MCIA complex would contain the ACAD9–ECSIT subcomplex with NDUFAF1 and ND2. We performed an *AlphaFold-Multimer* prediction (Jumper *et al.*, 2021 ▸; Evans *et al.*, 2022 ▸) of two copies each of human ACAD9 and ECSIT with a single copy of NDUFAF1 and ND2. A well connected structure was predicted for this multi-subunit complex (Fig. 6 ▸ *b*), showing the ACAD9 dimer and its interaction with the ECSIT fragment 322–334 (McGregor *et al.*, 2023 ▸) and the ND2–NDUFAF1 subcomplex similar to that in *Y. lipolytica* (Schiller *et al.*, 2022 ▸) (Fig. 6 ▸ *c*). The predicted model explains why all three proteins are necessary for complex formation (Formosa *et al.*, 2020 ▸), and also shows that both the N-terminal and C-terminal domains of ECSIT interact with NDUFAF1, but only the C-terminus interacts with ACAD9 (Giachin *et al.*, 2021 ▸). ECSIT makes extensive interactions with NDUFAF1 and with both copies of ACAD9. The helical N-terminal domain (residues 73–212), which was deleted in the cryo-EM studies (Giachin *et al.*, 2021 ▸; McGregor *et al.*, 2023 ▸), binds to NDUFAF1. The C-terminal domain (220–395) has a mixed α/β topology. It is inserted between NDUFAF1 and the ACAD9 dimer and includes a long loop between residues 311 and 354 (containing the 320–334 segment that is resolved in the cryo-EM map). This loop interacts closely with ACAD9 and NDUFAF1 and appears to be the main element keeping the complex together (Fig. 6 ▸ *b*). Another indication that the *AlphaFold* model is a good prediction is that both ACAD9 and NDUFAF1 have amphipathic helices that are positioned to serve as membrane anchors, similar to the tafazzin helix in the *Y. lipolytica* early P_P_ module intermediate (Figs. 6 ▸ *b* and 6 ▸ *c*). Interestingly, the long N-terminal amphipathic helix of human NDUFAF1 (residues 82–106) is truncated in *Y. lipolytica* (Figs. 6 ▸ *b* and 6 ▸ *c*). The ACAD9 helix 460–475 that folds under the C-terminal domain is disordered in the cryo-EM map (McGregor *et al.*, 2023 ▸; Fig. 6 ▸), which can be explained by the lack of a membrane in the preparation. Note, however, that one copy of ACAD9 overlaps with the position of the 45 kDa NDUFA10 subunit on the matrix side surface, which has been found as part of MCIA-containing assembly intermediates (Guerrero-Castillo *et al.*, 2017 ▸), so conformational changes would be necessary to insert this subunit. Further cryo-EM and functional studies will be needed to confirm this model for the MCIA complex, to show its presence *in vivo*, to reveal the role of the transmembrane assembly factors and to deduce further steps in building the P_P_ module.

## 4. The role of the assembly factor NDUFAF2

Apart from the 14 central subunits that are found in all species, mitochondrial complex I contains varying numbers of accessory subunits, which have been gained and lost at different evolutionary timepoints in different lineages (Gabaldón *et al.*, 2005 ▸; Elurbe & Huynen, 2016 ▸; Padavannil *et al.*, 2022 ▸). Three accessory subunits of the peripheral arm, NDUFS4, NDUFS6 and NDUFA12, are already found in complex I of α-proteobacteria, the group that contains the symbiont ancestor of mitochondria (Yip *et al.*, 2011 ▸). These subunits are conserved in mitochondrial complex I and mutations in their genes are associated with neurological diseases in humans (Kirby *et al.*, 2004 ▸; Ortigoza-Escobar *et al.*, 2016 ▸; Kahlhöfer *et al.*, 2021 ▸). The three subunits are located at the interface of the Q and N modules and appear to act in a late step of complex I assembly together with the assembly factor NDUFAF2 (Pereira *et al.*, 2013 ▸), which is a paralog of NDUFA12 (Gabaldón *et al.*, 2005 ▸; Ogilvie *et al.*, 2005 ▸). In a deletion mutant of NDUFS6 in *Y. lipolytica*, a complex I assembly intermediate accumulated that lacked not only NDUFS6 but also NDUFA12, and instead had assembly factor NDUFAF2 tightly bound (Kmita *et al.*, 2015 ▸). This strongly suggests that the assembly factor NDUFAF2 is replaced by its paralog NDUFA12 during complex I maturation.

**Figure 7:**
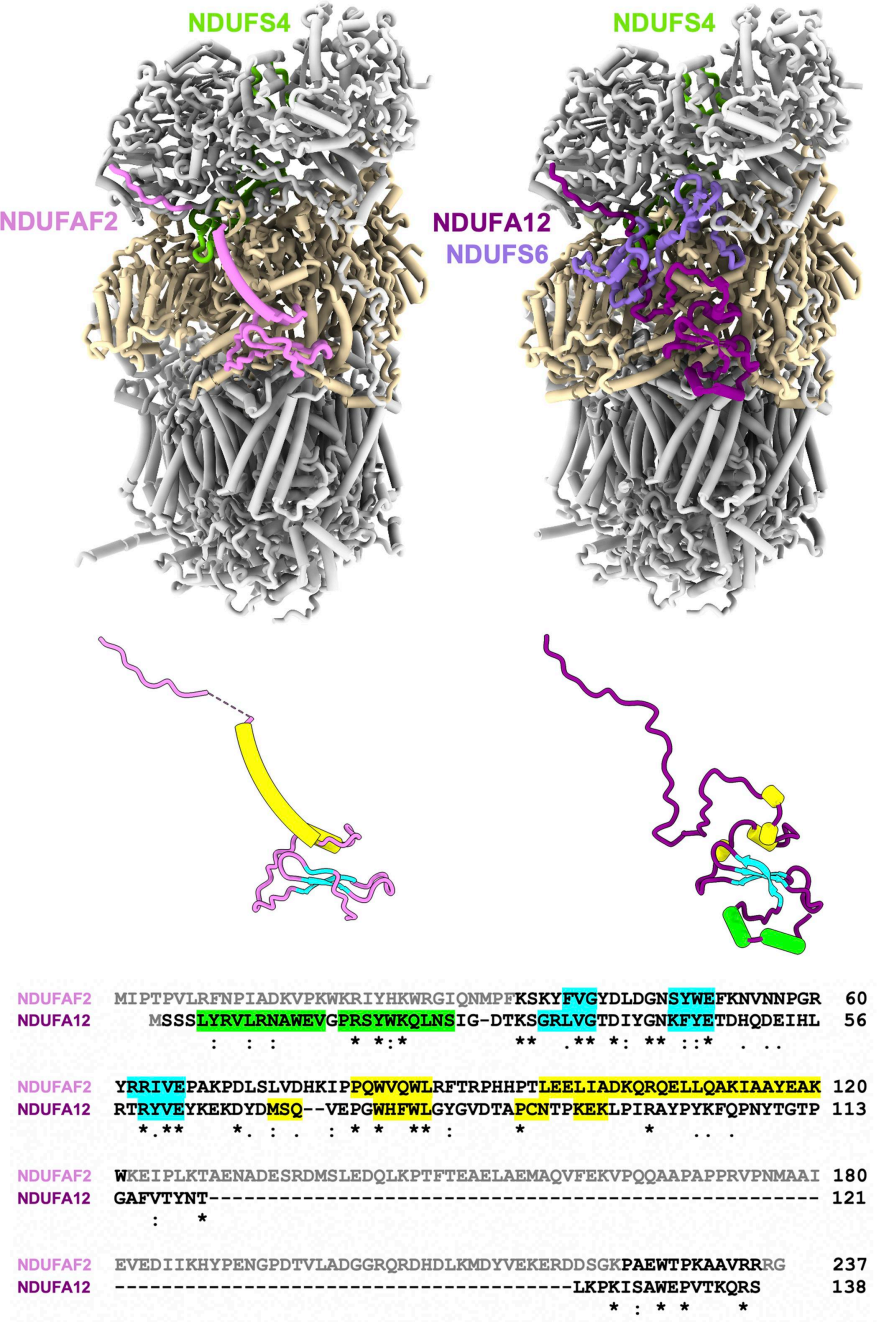
The role of NDUFAF2 in complex I assembly in *Y. lipolytica*. Left: the assembly intermediate from the *ndufs6*Δ strain (PDB entry 6rfq). The assembly factor NDUFAF2 (violet) is bound to the Q module (wheat) instead of NDUF12; NDUFS4 (green) is already in place. Right: in mature complex I (PDB entry 7o71), NDUFA12 (dark magenta) has replaced the assembly factor NDUFAF2, and NDUFS6 (medium purple) covers its C-terminus. Below: structure comparison and sequence alignment of NDUFAF2 and NDUFA12. β-Strands are shown in cyan, the N-terminal helices of NDUFA12 in lime and the other helices in yellow. The disordered N-terminus and 100-residue insert of NDUFAF2 are indicated in grey.

In order to determine the molecular function of NDUFAF2, the assembly intermediate from the *ndufs6*Δ strain was purified and studied by cryo-EM (Parey *et al.*, 2019 ▸). The structure at 3.3 Å resolution (Fig. 7 ▸) reveals the binding of assembly factor NDUFAF2 at the position of the NDUFA12 subunit and is otherwise very similar to mature complex I. The N-terminal domain of NDUFAF2 is bound to the Q module just above the membrane surface and has a similar fold to NDUFA12 (Fig. 7 ▸). However, the two short α-helices at the N-terminus of NDUFA12 that lie on the membrane surface and shape the lipid environment (Parey *et al.*, 2019 ▸) are unresolved in NDUFAF2, as are the lipids. The middle section of NDUFAF2 forms a long α-helix, whereas the equivalent part of NDUFA12 folds into an extended loop. The α-helix of NDUFAF2 overlaps with the binding site of NDUFS6, showing why this subunit is needed to dislodge NDUFAF2. The next ∼100 residues of NDUFAF2 are unresolved in the map and thus are probably disordered. Finally, the last 12 residues at the C-terminus of NDUFAF2 are bound to the N-module subunit NDUFS1 in an extended conformation, similar to the homologous C-terminus of NDUFA12 in mature complex I (Fig. 7 ▸). Thus, the following order of events is suggested. NDUFAF2 binds to an assembly intermediate of the Q module. The C-terminal stretch and probably also the middle domain that is later disordered support the attachment of the N module. Subsequently, NDUFA12 and NDUFS6 replace NDUFAF2, completing the formation of mature complex I. NDUFA12 is the cornerstone in the array of amphipathic helices at the interface of the P and Q modules (Parey *et al.*, 2019 ▸). Only after completion of this structure a functional access pathway for ubiquinone to its active site is formed. Thus, another role of transiently bound NDUFAF2 is likely to be the prevention of premature electron transfer and the formation of reactive oxygen species (ROS) in an incomplete complex I. In agreement with this, no substrate is found in the ubiquinone-binding cavity of the assembly intermediate, and quantitative mass spectrometry also shows only small amounts of bound ubiquinone (Parey *et al.*, 2019 ▸). Our present view of the assembly pathway of complex I in *Y. lipolytica* is summarized in Fig. 4 ▸.

NDUFS4, the third of the accessory subunits present in α-proteobacteria, is already in its final position in the cryo-EM structure of the ΔNDUFS6 assembly intermediate. NDUFS4 consists of a globular domain at the interface of the Q and N modules and a C-terminal coil extending up the N module. In humans, mutations in the NDUFS4 subunit cause Leigh syndrome, a serious neurological disorder (Ortigoza-Escobar *et al.*, 2016 ▸), and *Ndufs4* ^−/−^ mice develop similar symptoms (van de Wal *et al.*, 2022 ▸). A cryo-EM map of *Y. lipolytica ndufs4*Δ complex I contains all of the other subunits and shows only minor structural rearrangements around the groove created by the absence of NDUFS4 (Parey *et al.*, 2019 ▸). Analysis of the complex I surface showed that iron–sulfur clusters N3 and N1b were more exposed to solvent than in the wild type, explaining the increased ROS formation and the decreased electron-transfer activity of the mutant complex (Kahlhöfer *et al.*, 2017 ▸). Notably, a recent cryo-EM structure of mouse *Ndufs4* ^−/−^ complex I (Yin *et al.*, 2024 ▸) shows considerable differences from the mature complex. The complex was unstable and tended to lose the N module after purification. In order to allow high-resolution cryo-EM analysis, it had to be stabilized by cross-linking. The major class of particles did not contain either NDUFAF2 or NDUFA12 and showed a disordered N-terminal domain of NDUFS6. A minor class had the assembly factor NDUFAF2 still bound and was lacking NDUFS6. These observations confirm the assembly pathway deduced from the *Y. lipolytica* mutants (Parey *et al.*, 2019 ▸) and the role of NDUFAF2 in attaching the N module. However, in mice the absence of NDUFS4 has much more serious consequences for complex I function than in yeast and leads to a severe Leigh-like phenotype, probably due to the absence of NDUFA12 (Adjobo-Hermans *et al.*, 2020 ▸; Yin *et al.*, 2024 ▸). Unlike in yeast, in mice the absence of NDUFS4 appears to hinder the replacement of NDUFAF2 by NDUFA12. The question arises as to what causes this species difference. A possible answer lies in the N-terminal domain of NDUFS6, which has a different fold in the two species and is ∼50% larger in *Y. lipolytica* (58 versus 40 residues). It is conceivable that the smaller domain in mouse is less efficient in dislodging the assembly factor and/or in recruiting NDUFA12 in the distorted complex lacking NDUFS4. A low abundance of NDUFA12 was observed in these cells (Adjobo-Hermans *et al.*, 2020 ▸), possibly because the excess of free NDUFA12 was degraded.

## 5. Complex I assembly in plants

**Figure 8:**
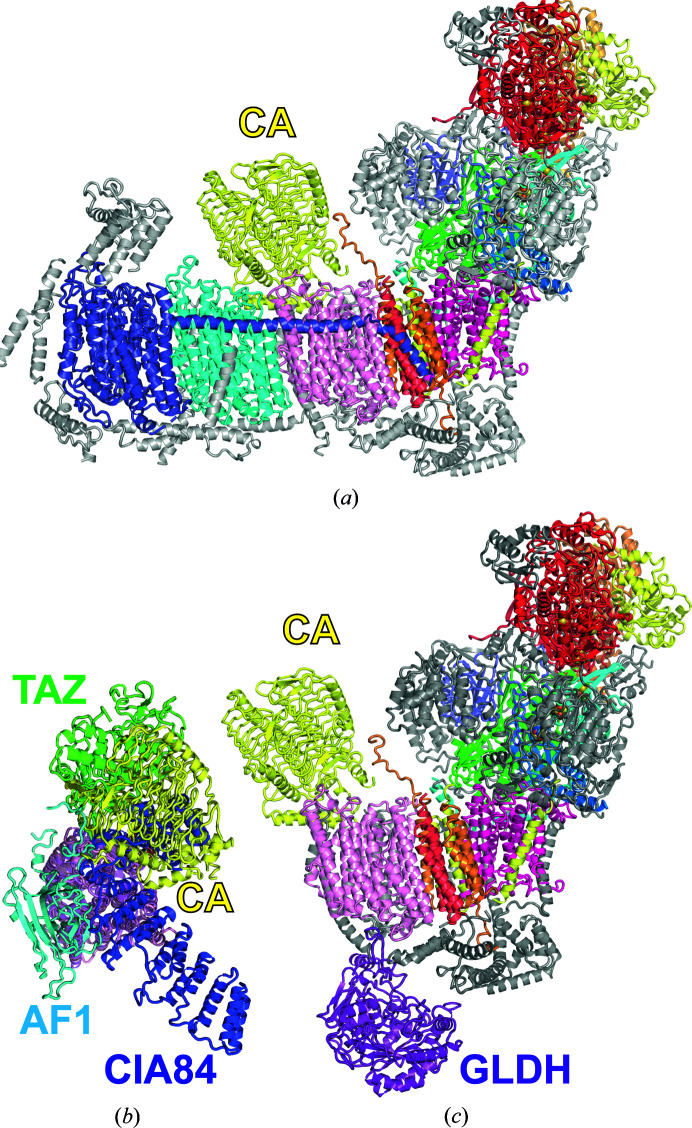
The assembly intermediate of plant complex I. (*a*) Side view of complex I from *Brassica oleracea* (PDB entry 7a23); central subunits are coloured as in Fig. 1 ▸, with the γ-carbonic anhydrase domain (CA) in yellow and all other accessory subunits in grey. (*b*) View from the matrix side of the early P_P_ module intermediate of *Y. lipolytica* complex I (compare with Fig. 2 ▸ *a*) overlaid with ND2 and the CA domain of the plant assembly intermediate shown in (*c*). The positions of CIA84 and CA show substantial overlap. (*c*) Side view of the complex I assembly intermediate (PDB entry 7a24) with GLDH (purple).

The structures of plant complex I (Soufari *et al.*, 2020 ▸; Klusch *et al.*, 2021 ▸) and of complex I in a I + III_2_ supercomplex (Klusch *et al.*, 2023 ▸; Maldonado *et al.*, 2023 ▸) have been determined by cryo-EM at up to 2 Å resolution. As a distinctive feature, complex I from plants has a prominent γ-carbonic anhydrase (CA) domain that is attached to the matrix side of the membrane arm (Fig. 8 ▸ *a*). The function of the CA domain is under debate (Braun & Klusch, 2024 ▸). It has been proposed that the formation of hydrogen carbonate from CO_2_ released in the mitochondrion is relevant to support carbon fixation in the Calvin–Benson cycle of chloroplasts. In addition, the position of the CA domain near the surface of the membrane arm could be important for the supply of protons for redox-linked proton translocation by complex I. It is interesting to note that the CA domain is added early in assembly (Ligas *et al.*, 2019 ▸) and it overlaps with the position of assembly factor CIA84 in fungal complex I (Fig. 8 ▸ *b*) and with the accessory subunit NDUFA10 in metazoa. It has been shown that CA emerged as a component of complex I early in evolution and has consequently been lost in opisthokonts (fungi and metazoa; Gawryluk & Gray, 2010 ▸). Deletion of one of the associated CA genes causes a severe complex I assembly defect (Perales *et al.*, 2005 ▸). Therefore, an additional role in complex I assembly seems plausible. The importance of another enzyme complex, l-galactono-1,4-lactone dehydro­genase (GLDH), for the assembly of plant complex I has been known for some time (Pineau *et al.*, 2008 ▸; Schertl *et al.*, 2012 ▸; Schimmeyer *et al.*, 2016 ▸), and the structure of an assembly intermediate with GLDH bound was recently determined by cryo-EM (Soufari *et al.*, 2020 ▸). GLDH sits on the intermembrane space side of a truncated membrane arm that lacks the P_D_ module (Fig. 8 ▸ *c*). The N-terminal tail of GLDH interacts with the ND2, NDUFC2 and NDUFS5 subunits. GLDH itself and the distorted NDUFC2 block the docking of the P_D_ module via the plant-specific accessory subunit P1. P1 appears to functionally replace the NDUFB5 subunit (Soufari *et al.*, 2020 ▸; Meyer *et al.*, 2022 ▸) and therefore the interlocking of the P_D_ and P_P_ modules in plants appears to occur by a similar mechanism as in fungi (Schiller *et al.*, 2022 ▸). Since the enzymatic activity of GLDH seems to be dispensable for complex I maturation (Schimmeyer *et al.*, 2016 ▸), its role is probably to stabilize the assembly intermediate and to prevent premature association with the P_D_ module (Soufari *et al.*, 2020 ▸). The mechanism by which GLDH is released is as yet unknown.

## 6. Conclusions and outlook

Cryo-EM is now the method of choice for the structure determination of large macromolecular complexes. The major challenges in the investigation of assembly processes are that the intermediates are typically present in small quantities and have limited stability. However, cryo-EM requires only micrograms of purified protein and advanced classification techniques allow the analysis of samples with compositional as well as conformational heterogeneity. In the best case, successive assembly steps can be identified in a complex sample on the same grid.

Our studies of complex I assembly in the yeast *Y. lipolytica* have made use of the genetic accessibility of this species and have shed light on the roles of assembly factors in the biogenesis of the largest complex of the mitochondrial respiratory chain. The assembly factor NDUFAF1 arrests the central subunit ND3 in an assembly-competent conformation and potentially protects its cysteine from oxidation. The assembly factor NDUFAF2 helps to join the N and Q modules and prevents the access of ubiquinone to the immature complex. Both assembly factors are highly conserved and also exist in mammalian mitochondria. NDUFAF2 has been shown to play a similar role in mouse mitochondria as in yeast, although the effects of mutations are more severe in mice. Mammalian NDUFAF1 has not yet been investigated. A cryo-EM structure of two other MCIA components, ACAD9 and ECSIT, indicates that ECSIT functions to remove the FAD cofactor from ACAD9 and to change it from an FAO enzyme to a complex I assembly factor. However, the function of these proteins in assembly is still not clear.

Cryo-EM analysis of the early P_P_ module intermediate also led to the discovery of its transient association with the cardiolipin-remodelling enzyme tafazzin. We hypothesize that this provides a supply of mature cardiolipin near the location of active protein assembly. Although this association appears to be specific for *Y. lipolytica*, in other species tafazzin has been shown to be linked to large protein assemblies in the inner mitochondrial membrane; however, their identity is unclear and remains to be investigated.

The first cryo-EM studies of the assembly process of mitochondrial complex I have already yielded many insights and unexpected findings. However, many aspects of this convoluted process still remain enigmatic. Not only are the structure, role and function of most assembly factors still unknown, but new assembly factors continue to be discovered. Cryo-EM will be indispensable for gaining a comprehensive understanding of the structural basis of complex I assembly in the future.

## Figures and Tables

**Table 1:** Complex I assembly factors in *Homo sapiens* and *Yarrowia lipolytica* Abbreviations: *Hs*, *Homo sapiens*; *Yl*, *Yarrowia lipolytica*; LYRM, leucine–tyrosine–arginine motif-containing protein; NUBPL, nucleotide-binding protein-like; TIMMDC, translocase of inner mitochondrial membrane domain-containing protein; TMEM, transmembrane protein; COA, cytochrome *c* oxidase assembly factor; ACAD, acyl-CoA dehydrogenase family member; ECSIT, evolutionary conserved signalling intermediate in Toll pathway; CIA, complex I intermediate-associated protein; SFXN, sideroflexin; FOXRED, FAD-dependent oxidoreductase domain-containing protein; DMAC, distal membrane-arm assembly complex protein.

Module	Assembly factor	*Hs*	*Yl*	Structural data	Alternative names	References
N	NDUFAF2	Yes	Yes	*Yl*, 6rfq	N7BML, NDUFA12L, B17.2L, mimitin	Ogilvie *et al.* (2005 ▸), Parey *et al.* (2019 ▸)
N	LYRM2	Yes	Yes			Dibley *et al.* (2020 ▸)
N/Q	NUBPL	Yes	Yes		IND1	Bych *et al.* (2008 ▸), Friederich *et al.* (2020 ▸)
Q	NDUFAF3	Yes	Yes		C3orf60	Saada *et al.* (2009 ▸), Murari *et al.* (2021 ▸)
Q	NDUFAF4	Yes	No		C6orf66	Saada *et al.* (2008 ▸), Baertling *et al.* (2017 ▸), Murari *et al.* (2021 ▸)
Q	NDUFAF5	Yes	Yes		C20orf7	Sugiana *et al.* (2008 ▸), Chen *et al.* (2023 ▸)
Q	NDUFAFQ	Yes	No		preY, PYURF	Rensvold *et al.* (2022 ▸)
Q	NDUFAF7	Yes	Yes		C2orf56, PRO1853	Zurita Rendón *et al.* (2014 ▸), Rhein *et al.* (2013 ▸)
Q	NDUFAF8	Yes	Yes		C17orf89	Floyd *et al.* (2016 ▸), Peker *et al.* (2023 ▸)
P_P_/Q	TIMMDC1	Yes	No		C3orf1	Guarani *et al.* (2014 ▸), Wang *et al.* (2021 ▸)
P_P_	NDUFAF6	Yes	Yes		C8orf38	Pagliarini *et al.* (2008 ▸), Lemire (2017 ▸)
P_P_/MCIA	NDUFAF1	Yes	Yes	*Yl*, 7zkq, 7zkp	CIA30, CGI-65	Küffner *et al.* (1998 ▸), Schiller *et al.* (2022 ▸)
P_P_/MCIA	TMEM186	Yes	No		C16orf51	Guerrero-Castillo *et al.* (2017 ▸), Formosa *et al.* (2020 ▸)
P_P_/MCIA	TMEM126B	Yes	No		HT007	Heide *et al.* (2012 ▸), Fuhrmann *et al.* (2018 ▸)
P_P_/MCIA	COA1	Yes	No		C7orf44, MITRAC15	Guerrero-Castillo *et al.* (2017 ▸), Wang, Richter-Dennerlein *et al.* (2020 ▸)
P_P_/MCIA	ACAD9	Yes	Yes	*Hs* †		Nouws *et al.* (2010 ▸), McGregor *et al.* (2023 ▸)
P_P_/MCIA	ECSIT	Yes	No	*Hs* †		Vogel, Janssen *et al.* (2007 ▸), McGregor *et al.* (2023 ▸)
P_P_	CIA84	No	Yes	*Yl*, 7zkq, 7zkp	B11O8.100, NCU01006	Küffner *et al.* (1998 ▸), Schiller *et al.* (2022 ▸)
P_P_	SFXN4	Yes	No		BCRM1	Jackson *et al.* (2022 ▸)
P_D_	TMEM70	Yes	No			Sánchez-Caballero *et al.* (2020 ▸), Carroll *et al.* (2021 ▸)
P_D_	TMEM126A	Yes	No		OPA7	D’Angelo *et al.* (2021 ▸), Formosa *et al.* (2021 ▸)
P_D_	FOXRED1	Yes	Yes		FP634	Fassone *et al.* (2010 ▸), Formosa *et al.* (2015 ▸)
P_D_	DMAC2	Yes	No		ATP5SL	Stroud *et al.* (2016 ▸)
P_D_	DMAC1	Yes	No		TMEM261	Stroud *et al.* (2016 ▸), Horlbeck *et al.* (2018 ▸)

† Giachin *et al.* (2021 ▸), McGregor *et al.* (2023 ▸).
